# Levels of selected trace elements in Scots pine (*Pinus sylvestris* L.), silver birch (*Betula pendula* L.), and Norway maple (*Acer platanoides* L.) in an urbanized environment

**DOI:** 10.1007/s10661-016-5600-0

**Published:** 2016-09-30

**Authors:** Milena Kosiorek, Beata Modrzewska, Mirosław Wyszkowski

**Affiliations:** Department of Environmental Chemistry, University of Warmia and Mazury in Olsztyn, Plac Łódzki 4, 10-727 Olsztyn, Poland

**Keywords:** Trace elements, Trees, Needles, Leaves, Bark, Soil

## Abstract

The aim of the study was to determine the concentrations of selected trace elements in needles and bark of Scots pine (*Pinus sylvestris* L.), leaves and bark of silver birch (*Betula pendula* L.), and Norway maple (*Acer platanoides* L.), as well as in the soil in which the trees grew, depending on their localization and hence the distribution of local pollution sources. The content of trace elements in needles of Scots pine, leaves of silver birch, and Norway maple and in bark of these trees depended on the location, tree species, and analyzed organ. The content of Fe, Mn, and Zn in needles, leaves, and bark of the examined tree species was significantly higher than that of the other elements. The highest average content of Fe and Mn was detected in leaves of Norway maple whereas the highest average content of Zn was found in silver birch leaves. The impact of such locations as the center of Olsztyn or roadside along Road 51 on the content of individual elements tended to be more pronounced than the influence of the other locations. The influence of the sampling sites on the content of trace elements in tree bark was less regular than the analogous effect in needles and leaves. Moreover, the relevant dependences were slightly different for Scots pine than for the other two tree species. The concentrations of heavy metals determined in the soil samples did not exceed the threshold values set in the Regulation of the Minister for the Environment, although the soil along Road 51 and in the center of Olsztyn typically had the highest content of these elements. There were also significant correlations between the content of some trace elements in soil and their accumulation in needles, leaves, and bark of trees.

## Introduction

For countless centuries in the past, environmental pollution had originated from natural sources. As civilizations began to emerge and grow, anthropogenic pollutants played an increasingly important role. These include heavy metals, which are mostly generated by such industries as mining and metallurgy, but also waste processing, energy generation, combustion of fuels in transport, agriculture (plant protection chemicals, mineral, and organic fertilizers), and by households (Alagić et al. 2013, Kopponen et al. 2001, Sawidis et al. 2011, Staszewski et al. 2012). All these factors contribute to the emission of pollutants in the form of gases, aerosols, and liquids, which subsequently, through dry and wet deposition, accumulate in all ecosystems (Kozlov 2005, Sawidis et al. 2011, Staszewski et al. 2012). Among the sites most distinctly exposed to heavy metal contamination are urban areas (Malinowska 2010, Sun et al. 2009). The spatial management of towns should envisage tree planting for esthetic, climatic, and health-related reasons (Kozik et al. 2014).

The content of trace elements depends on local climatic conditions, type of habitat, level of groundwater, activity of soil-dwelling microoorganisms, tree species, its developmental stage, organ, and its content of nutrients (Hagen-Thorn et al. 2004, Hellsten et al. 2013, Modrzewska and Wyszkowski 2014, Rademacher 2005, Roitto et al. 2005). Because heavy metals accumulate differently in various tree organs, determination of the degree of pollution is most frequently achieved by analyzing the composition of bark, leaves, needles, shoots, buds, and roots (Alagić et al. 2013, Kirchner et al. 2008, Piczak et al. 2003). To the highest extent, the content of heavy metals in these tree organs is affected by the structure of a given organ and the contamination of atmospheric air. Owing to its porosity, bark enables us detect long-term contamination with trace elements; in contrast, pollutants that settle down on leaves can be rinsed off by rains or dispersed by winds (Kirchner et al. 2008, Sawidis et al. 2011). Trees are distinguished by various degrees of tolerance to environmental pollution. Some are more tolerant to elevated contents of heavy metals and do not present visible disease symptoms (Alagić et al. 2013, Ivanov et al. 2011). The typical consequences of increased levels of heavy metals are dysregulated uptake of elements essential for the plant’s proper growth and development, decelerated germination of seeds and growth of the root system, retarded growth of tree biomass, and inhibited photosynthetic processes (Ivanov et al. 2011, Malinowska 2010). The resistance of trees to environmental pollution, their popularity, the type and size of leaves, the type of bark, and root system were crucial when selecting test tree species (Alagić et al. 2013, Parzych and Sobisz 2012).

The above considerations have encouraged us to undertake a study in order to determine concentrations of selected trace elements in needles and bark of Scots pine (*Pinus sylvestris* L.), leaves and bark of silver birch (*Betula pendula* L.), and Norway maple (*Acer platanoides* L.), as well as in the soil in which the trees grew, depending on their localization and hence the distribution of local pollution sources.

## Material and methods

### Sampling

**Fig. 1:**
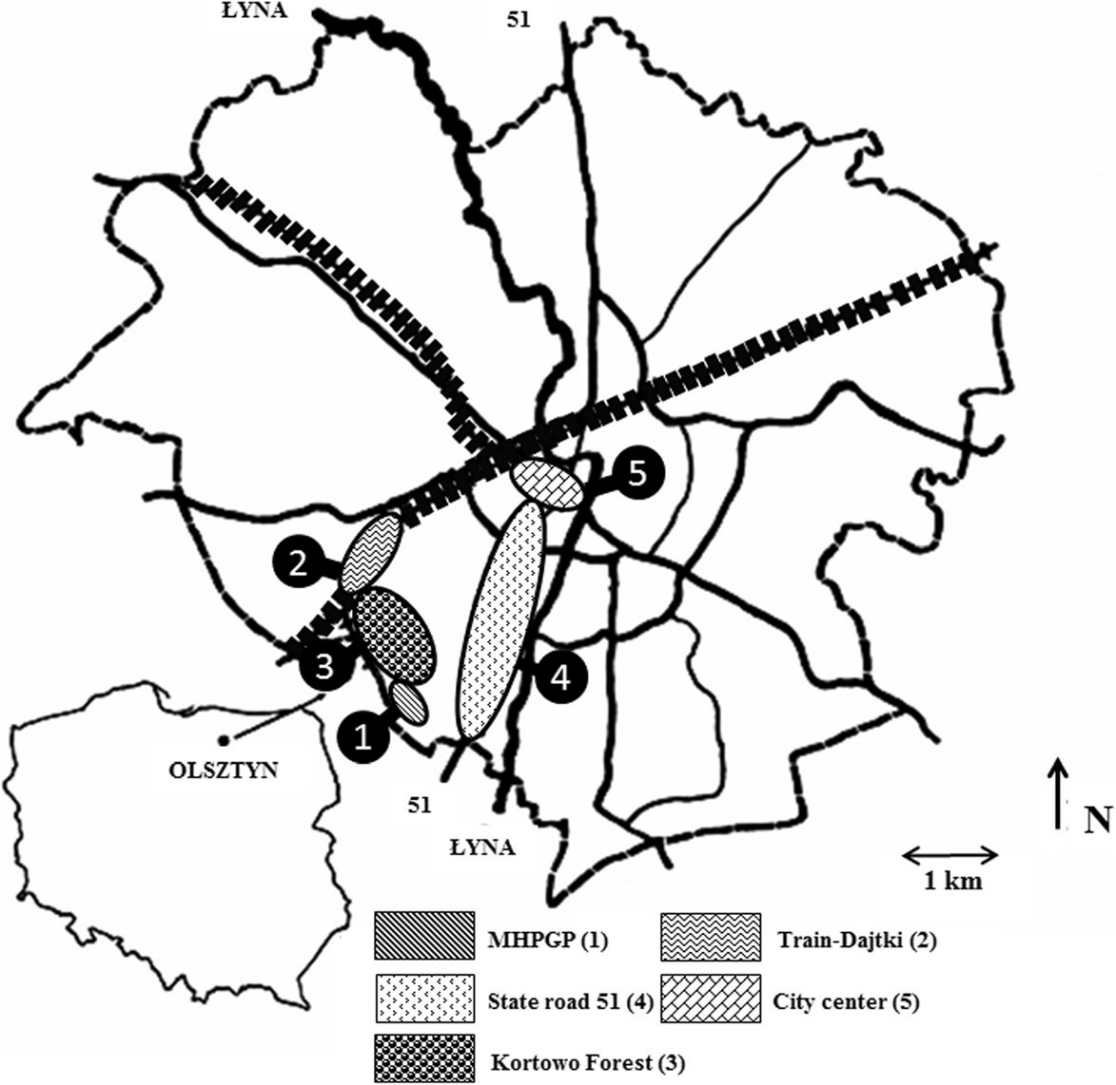
Map presenting the distribution of sampling sites in Olsztyn

The research covered five sites located within the town limits of Olsztyn (northeastern Poland): near the Municipal Heat Power Generation Plant—MHPGP, along a railway track in the housing estate called Dajtki, in Kortowo Forest, at State Road 51 leading out of the town and in the town’s center between 22 Stycznia Street and Jedności Słowiańskiej Square. The detailed information about localization of these sites are presented in Fig. 1 and in Table 1. In each location, specimens of three tree species: Scots pine *Pinus sylvestris* L.), silver birch (*Betula pendula* L.), and Norway maple (*Acer platanoides* L.) were selected. The trees were approximately the same age and in the stage of physiological dormancy. At each site, five trees of the same species were selected, and five samples consisting of 50 g of needles, leaves, and bark were taken from each tree. The needles and leaves were collected from the central height of a tree, from the middle length of a branch, while bark was samples at a breast height of 130 cm above the ground, leaving the phloem intact. The samples obtained from each sampling point were aggregated into a collective, representative sample weighing 250 g. Meanwhile, soil samples were taken from the surface soil layer, using an Egner sampler. At each place, four soil samples under of edge of canopy of each tree (0–15 cm soil depth) were collected, and four samples consisting of 60 g of soil were taken from each tree.

**Table 1:** The localisation of research sites in Olsztyn

Site	Coordinates in degrees, minutes, and seconds
Municipal Heat Power Generation Plant—MHPGP	53° 44′ 50.6″ N–20° 26′ 44.0″ E
Train–Dajtki	53° 45′ 38.9″ N–20° 26′ 04.5″ E
Kortowo Forest	53° 45′ 13.9″ N–20° 26′ 27.0″ E
State Road 51	53° 46′ 04.6″ N–20° 28′ 01.5″ E
Town’s center between 22 Stycznia Street and Jedności Słowiańskiej Square	53° 46′ 35.7″ N–20° 28′ 57.2″ E (22 Stycznia Street) 53° 46′ 37.3″ N–20° 28′ 44.1″ E (Jedności Słowiańskiej Square)

### Methods of analyses

The collected plant material was dried at 60 °C and milled. Ready plant samples were wet-mineralized in concentrated nitrogen(V) acid (HNO_3_ analytical grade) at a concentration of 1.40 g cm^−3^, in Teflon HP 500 vessels placed in a MARS 5 microwave oven (Microwave Accelerated Reaction System, CEM Corporation, USA). Soil was dried and passed through a sieve with the mesh net size of 1 mm, after which it was mineralized in concentrated nitrogen(V) acid in the same apparatus as applied for the mineralization of plant material. The plant and soil material prepared as explained above was submitted to determination of the total content of microelements: Pb, Cd, Cr, Ni, Mn, Zn, Cu, Fe, and Co, using flame atomic absorption spectrometry (FAAS) in an air-acetylene flame, on a spectrometer SpectrAA 240FS (VARIAN, Australia), according to the US-EPA3051 method (1994). Deionized water of the conductivity below 0.05 μS cm^−1^ was used for determinations. To verify the results, plant certified reference materials NCS ZC 73030 originating from China National Analysis Center for Iron & Steel 2014 and AGH S-1 Polish Soil as well as reference solutions by Fluka: Pb 16595, Cd 51994, Cr 02733, Ni 42242, Mn 63534, Zn 18827, Cu 38996, Fe 16596, and Co 119785.0100 were used. The research results were processed statistically with two-factorial ANOVA and PCA, both available in a Statistica software package (StatSoft Inc. 2014). Moreover, the content of P, K, Na, Ca, and Mg was determined in needles, leaves, and bark of the trees, and these results were reported in an earlier paper (Modrzewska et al. 2016).

## Results

### Content of trace elements in needles, leaves, and bark of trees

The research demonstrated that the content of lead, cadmium, chromium, nickel, manganese, zinc, copper, iron, and cobalt in needles of Scots pine and in leaves of silver birch and Norway maple as well as in the bark of these tree species depended on the location and species of individual trees (Tables 2–4).

**Table 2:** Content of lead, cadmium, and chromium in the needles and bark of Scots pine (*Pinus sylvestris* L.), leaves and bark of silver birch (*Betula pendula* L.), and Norway maple (*Acer platanoides* L.), average ± standard deviation in milligram per kilogram of dry matter

	Needles/leaves			Bark		
	Scots pine	Silver birch	Norway maple	Scots pine	Silver birch	Norway maple
Lead						
MHPGP	0.097 ± 0.011	0.156 ± 0.007	0.127 ± 0.013	0.098 ± 0.004	0.163 ± 0.004	0.143 ± 0.004
Train–Dajtki	0.143 ± 0.001	0.169 ± 0.001	0.145 ± 0.000	0.092 ± 0.002	0.097 ± 0.001	0.131 ± 0.000
Kortowo Forest	0.114 ± 0.015	0.160 ± 0.009	0.187 ± 0.012	0.138 ± 0.008	0.155 ± 0.007	0.153 ± 0.018
State Road 51	0.154 ± 0.004	0.171 ± 0.003	0.152 ± 0.002	0.119 ± 0.004	0.139 ± 0.000	0.194 ± 0.003
City center	0.132 ± 0.004	0.192 ± 0.008	0.187 ± 0.001	0.155 ± 0.012	0.154 ± 0.008	0.170 ± 0.018
Average	0.128	0.170	0.160	0.120	0.142	0.158
LSD	a, 0.009; b, 0.007; a·b, 0.016			a, 0.010; b, 0.008; a·b, 0.018		
Cadmium						
MHPGP	0.032 ± 0.000	0.034 ± 0.003	0.034 ± 0.003	0.018 ± 0.000	0.007 ± 0.003	0.018 ± 0.000
Train–Dajtki	0.029 ± 0.003	0.038 ± 0.003	0.036 ± 0.000	0.014 ± 0.006	0.027 ± 0.000	0.029 ± 0.003
Kortowo Forest	0.034 ± 0.003	0.036 ± 0.000	0.043 ± 0.003	0.029 ± 0.003	0.023 ± 0.000	0.043 ± 0.003
State Road 51	0.027 ± 0.000	0.005 ± 0.000	0.005 ± 0.000	0.029 ± 0.003	0.032 ± 0.000	0.034 ± 0.003
City center	0.007 ± 0.003	0.007 ± 0.003	0.005 ± 0.000	0.034 ± 0.003	0.032 ± 0.006	0.041 ± 0.006
Average	0.026	0.024	0.024	0.025	0.024	0.033
LSD	a, 0.003; b, 0.002; a·b, 0.005			a, 0.004; b, 0.003; a·b, 0.008		
Chromium						
MHPGP	0.351 ± 0.013	0.308 ± 0.013	0.274 ± 0.000	0.323 ± 0.000	0.332 ± 0.013	0.308 ± 0.030
Train–Dajtki	0.326 ± 0.022	0.345 ± 0.047	0.296 ± 0.056	0.357 ± 0.004	0.348 ± 0.043	0.329 ± 0.026
Kortowo Forest	0.302 ± 0.004	0.320 ± 0.004	0.308 ± 0.030	0.381 ± 0.039	0.372 ± 0.035	0.320 ± 0.013
State Road 51	0.335 ± 0.035	0.345 ± 0.022	0.372 ± 0.009	0.369 ± 0.013	0.354 ± 0.026	0.329 ± 0.035
City center	0.363 ± 0.022	0.387 ± 0.004	0.433 ± 0.035	0.348 ± 0.009	0.323 ± 0.009	0.326 ± 0.030
Average	0.335	0.341	0.337	0.356	0.346	0.323
LSD	a, 0.033; b, 0.025; a·b, 0.057			a, 0.031; b, 0.024; a·b, 0.054		

LSD for a, place of sampling; b, tree species; a·b, interaction; significant at *P* ≤ 0.05

**Fig. 2:**
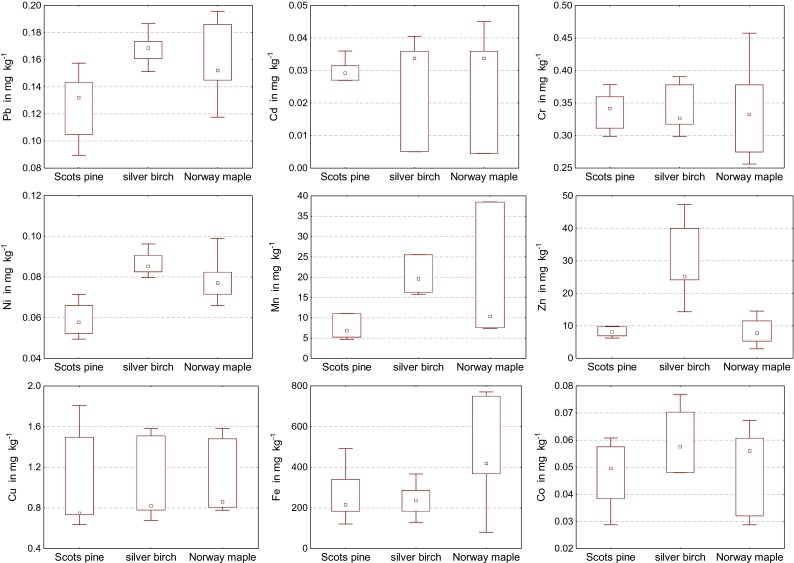
The average content of lead, cadmium, chromium, nickel, manganese, zinc, copper, iron and cobalt in the needles of Scots pine (*Pinus silvestris* L.), leaves of silver birch (*Betula pendula* L.) and Norway maple (*Acer platanoides* L.), (in milligram per kilogram of dry matter)

**Fig. 3:**
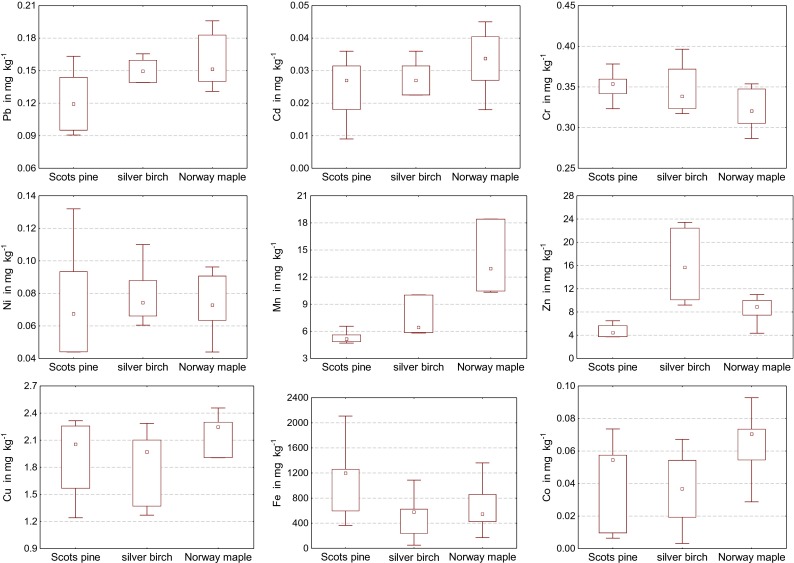
The average content of lead, cadmium, chromium, nickel, manganese, zinc, copper, iron and cobalt in the bark of Scots pine (*Pinus silvestris* L.), silver birch (*Betula pendula* L.) and Norway maple (*Acer platanoides* L.), (in milligram per kilogram of dry matter)

The average content of manganese and zinc in needles and leaves of the analyzed trees was much higher than in their bark (Figs. 2 and 3) A reverse tendency emerged for copper and iron, while the concentrations of the remaining elements were similar in both organs. Out of all the analyzed heavy elements, iron, followed by manganese and zinc, was distinguished by the highest average content in needles, leaves, and bark of the examined tree species. The highest average content of iron and manganese, i.e., 473.5 and 166.282 mg kg^−1^, respectively, was detected in leaves of Norway maple, whereas the highest zinc content (30.150 mg kg^−1^) was determined in leaves of silver birch (Tables 3 and 4). The average content of iron and manganese in leaves of Norway maple was 2-fold (Fe) and 10-fold (Mn) higher than in needles of Scots pine. Leaves of silver birch contained about 3.5-fold more zinc than needles of Scots pine or leaves of Norway maple. The center of Olsztyn and the roadside along Road 51 were characterized by the highest concentration of iron in leaves of Norway maple (over 700.0 mg kg^−1^). Similar dependences were verified for silver birch, although the amounts of iron accumulated in leaves of this tree species were less than a half of the quantities in Norway maple leaves. Needles of Scots pine collected along the railway track in Dajtki and in the town center had distinctly more of this element (491.2 and 337.5 mg kg^−1^) than needles collected elsewhere. The impact of the town’s central location manifested itself as a slight increase in the content of lead in leaves of Norway maple and silver birch. The needles and leaves sampled in the center of Olsztyn contained higher amounts of cadmium and cobalt, while higher quantities of cadmium, but not cobalt, were found only in leaves of Norway maple and silver birch among the plant samples collected along Road 51. Elevated levels of manganese were detected in needles and leaves of all the three tree species growing in Kortowo Forest, especially in leaves of Norway maple (769.444 mg kg^−1^). The highest copper content and the lowest amount of lead were determined in needles and leaves of trees growing near the Municipal Heat Power Generation Plant (the MHPGP). The content of nickel and zinc was not closely correlated with the sampling sites or the presence of local pollution sources.

**Table 3:** Content of nickel, manganese, and zinc in the needles and bark of Scots pine (*Pinus silvestris* L.), leaves and bark of silver birch (*Betula pendula* L.), and Norway maple (*Acer platanoides* L.), average ± standard deviation in milligram per kilogram of dry matter

	Needles/leaves			Bark		
	Scots pine	Silver birch	Norway maple	Scots pine	Silver birch	Norway maple
Nickel						
MHPGP	0.062 ± 0.002	0.083 ± 0.004	0.072 ± 0.008	0.047 ± 0.004	0.077 ± 0.023	0.054 ± 0.014
Train–Dajtki	0.050 ± 0.000	0.083 ± 0.000	0.074 ± 0.012	0.056 ± 0.018	0.070 ± 0.002	0.072 ± 0.000
Kortowo Forest	0.054 ± 0.002	0.122 ± 0.053	0.087 ± 0.014	0.055 ± 0.016	0.069 ± 0.012	0.077 ± 0.027
State Road 51	0.063 ± 0.012	0.094 ± 0.004	0.085 ± 0.019	0.107 ± 0.035	0.087 ± 0.002	0.085 ± 0.008
City center	0.069 ± 0.004	0.085 ± 0.008	0.078 ± 0.006	0.098 ± 0.006	0.088 ± 0.031	0.083 ± 0.012
Average	0.059	0.093	0.079	0.073	0.078	0.074
LSD	a, 0.020; b, 0.015; a·b, 0.034			a, 0.022; b, 0.017; a·b, 0.037		
Manganese						
MHPGP	10.992 ± 0.082	24.881 ± 0.951	10.370 ± 0.081	4.920 ± 0.104	9.833 ± 0.290	12.940 ± 0.084
Train–Dajtki	4.732 ± 0.015	19.574 ± 0.641	7.453 ± 0.078	4.753 ± 0.074	5.981 ± 0.227	10.461 ± 0.016
Kortowo Forest	56.663 ± 1.350	314.255 ± 5.698	769.444 ± 6.923	5.247 ± 0.030	65.396 ± 2.606	373.874 ± 10.800
State Road 51	6.871 ± 1.274	16.436 ± 0.423	36.480 ± 2.895	5.343 ± 0.404	6.272 ± 0.639	18.060 ± 0.521
City center	5.236 ± 0.015	16.026 ± 0.298	7.662 ± 0.218	6.507 ± 0.092	5.927 ± 0.091	10.381 ± 0.065
Average	16.899	78.234	166.282	5.354	18.682	85.143
LSD	a, 3.080; b, 2.386; a·b, 5.335			a, 3.548; b, 2.748; a·b, 6.145		
Zinc						
MHPGP	9.503 ± 0.393	25.093 ± 0.057	3.376 ± 0.635	3.780 ± 0.064	23.417 ± 0.029	9.579 ± 0.657
Train–Dajtki	6.465 ± 0.321	46.986 ± 0.457	6.798 ± 2.205	6.223 ± 0.378	15.655 ± 0.928	4.542 ± 0.271
Kortowo Forest	7.883 ± 0.029	14.555 ± 0.328	14.540 ± 0.007	0.444 ± 0.057	21.868 ± 0.849	7.777 ± 0.478
State Road 51	9.841 ± 0.043	24.411 ± 0.421	11.042 ± 0.699	5.405 ± 0.378	9.851 ± 0.300	10.947 ± 0.079
City center	7.626 ± 1.078	39.703 ± 0.421	7.146 ± 0.100	4.456 ± 0.692	11.285 ± 2.955	8.867 ± 0.221
Average	8.264	30.150	8.581	4.062	16.415	8.342
LSD	a, 0.089; b, 0.691; a·b, 1.544			a, 1.100; b, 0.852; a·b, 1.905		

LSD for a, place of sampling; b, tree species; a·b, interaction; significant at *P* ≤ 0.05

**Table 4:** Content of copper, iron, and cobalt in the needles and bark of Scots pine (*Pinus silvestris* L.), leaves and bark of silver birch (*Betula pendula* L.), and Norway maple (*Acer platanoides* L.), average ± standard deviation in milligram per kilogram of dry matter

	Needles/leaves			Bark		
	Scots pine	Silver birch	Norway maple	Scots pine	Silver birch	Norway maple
Copper						
MHPGP	1.715 ± 0.130	1.567 ± 0.020	1.546 ± 0.050	1.256 ± 0.020	1.292 ± 0.030	1.278 ± 0.030
Train–Dajtki	1.496 ± 0.000	1.504 ± 0.010	1.475 ± 0.010	1.574 ± 0.010	2.238 ± 0.070	2.407 ± 0.070
Kortowo Forest	0.748 ± 0.000	0.784 ± 0.010	0.812 ± 0.030	2.111 ± 0.030	2.047 ± 0.080	2.259 ± 0.060
State Road 51	0.671 ± 0.050	0.819 ± 0.020	0.861 ± 0.000	2.287 ± 0.040	2.012 ± 0.090	2.280 ± 0.010
City center	0.734 ± 0.000	0.678 ± 0.000	0.791 ± 0.020	2.160 ± 0.200	1.532 ± 0.230	1.948 ± 0.060
Average	1.073	1.070	1.097	1.878	1.824	2.034
LSD	a, 0.050; b, 0.038; a·b, 0.086			a, 0.114; b, 0.088; a·b, 0.198		
Iron						
MHPGP	215.5 ± 7.3	154.2 ± 35.5	96.0 ± 22.0	390.4 ± 37.2	596.4 ± 7.3	172.1 ± 2.3
Train–Dajtki	491.2 ± 0. 6	227.1 ± 28. 2	376.5 ± 13.0	1198.4 ± 19.2	594.4 ± 45.1	455.0 ± 42.8
Kortowo Forest	121.9 ± 1.1	206.4 ± 32. 7	418.7 ± 15.2	613.9 ± 29.9	70.5 ± 28.7	545.4 ± 3.9
State Road 51	196.0 ± 19.1	327.5 ± 56.3	722.3 ± 68.2	1261.0 ± 3.9	1066.1 ± 30.4	1337.1 ± 33.8
City center	337.5 ± 5.1	296.0 ± 38.9	753.8 ± 4.5	2262.9 ± 219.7	327.1 ± 130.1	858.2 ± 4.5
Average	272.4	242.2	473.5	1145.3	530.9	673.5
LSD	a, 37.355; b, 28.935; a·b, 64.701			a, 86.913; b, 67.323; a·b, 150.539		
Cobalt						
MHPGP	0.059 ± 0.002	0.067 ± 0.014	0.056 ± 0.002	0.011 ± 0.002	0.006 ± 0.005	0.032 ± 0.005
Train–Dajtki	0.046 ± 0.011	0.070 ± 0.000	0.062 ± 0.007	0.008 ± 0.002	0.027 ± 0.011	0.054 ± 0.000
Kortowo Forest	0.050 ± 0.011	0.061 ± 0.005	0.062 ± 0.002	0.056 ± 0.002	0.037 ± 0.002	0.074 ± 0.000
State Road 51	0.050 ± 0.002	0.050 ± 0.002	0.035 ± 0.005	0.056 ± 0.002	0.061 ± 0.009	0.070 ± 0.005
City center	0.034 ± 0.007	0.011 ± 0.002	0.029 ± 0.000	0.074 ± 0.000	0.056 ± 0.011	0.083 ± 0.014
Average	0.048	0.052	0.049	0.041	0.037	0.063
LSD	a, 0.008; b, 0.006; a·b, 0.014			a, 0.008; b, 0.006; a·b, 0.014		

LSD for a, place of sampling; b, tree species; a·b, interaction; significant at *P* ≤ 0.05

The content of manganese and, especially, of iron in tree bark was demonstrably higher than that of the other trace elements (Tables 2, 3, and 4). The bark of Scots pine was distinguished by about 2-fold higher content of iron than the bark of silver birch or Norway maple (Fig. 3). The bark of Norway maple contained 16-fold more manganese and the bark of silver birch had 4-fold more zinc than the bark of Scots pine. The effect of a sampling site on the content of trace elements in tree bark was less regular than determined for leaves and needles and usually slightly different in the case of Scots pine than in the other tree species. The highest iron content (2262.9 mg kg^−1^) was detected in the bark of Scots pine in the center of Olsztyn, manganese (373.874 mg kg^−1^) in the bark of Norway maple in Kortowo Forest, and zinc (23.417 mg kg^−1^) in the bark of silver birch near the MHPGP. The bark of all tree species collected in the vicinity of Road 51 was found to have an elevated content of cobalt, while the bark samples collected in the center contained increased amounts of cadmium and cobalt. The Scots pine bark sampled in the center had the highest content of iron, lead, cadmium, manganese, nickel, and copper, while the samples originating from the Road 51 location had the highest nickel and copper content. The bark of Norway maple and silver birch growing along Road 51 had the highest content of iron, while the samples from Kortowo Forests were the most abundant in manganese. There were very small differences in concentrations of the remaining elements determined in the bark of the test trees.

### Content of trace elements in soils

When confronted with the regulation of the Minister for the Environment in Poland (2002), the concentrations of heavy metals determined in the soil samples taken at all the locations did not exceed the thresholds established for soils covered in trees or in shrubs (Table 5). Out of the five sampling sites, the soil along Road 51 and in the town center had a distinctly higher content of lead (37.50 and 54.70 mg kg^−1^), iron (7689.9 and 7106.5 mg kg^−1^), copper (32.60 and 36.60 mg kg^−1^), and cobalt (8.90 and 9.60 mg kg^−1^). The highest content of zinc (36.72 mg/kg) was found in the town center, and that of cadmium (0.25 mg kg^−1^) and chromium (24.20 mg kg^−1^) occurred in soil along Road 51, while the highest soil content of manganese (217.00 mg kg^−1^) was detected in Kortowo Forest. The soil collected in the forest was also distinguished by the lowest content of lead (18.85 mg kg^−1^), nickel (7.00 mg kg^−1^), and zinc (5.06 mg kg^−1^). The quantities of the analyzed elements in soil samples from the other sampling sites were only slightly varied.

**Table 5:** Content selected heavy metals in soil depending on the place of collection, in milligram per kilogram of soil, average ± standard deviation in milligram per kilogram of soil

	Pb	Cd	Cr	Ni	Mn	Zn	Cu	Fe	Co
MHPGP	25.05 ± 0.75	0.14 ± 0.06	21.00 ± 1.13	12.20 ± 2.26	152.30 ± 3.82	14.68 ± 0.42	22.30 ± 1.56	6421.8 ± 62.51	5.00 ± 0.28
Train–Dajtki	28.45 ± 2.05	0.22 ± 0.00	18.90 ± 0.42	14.80 ± 8.49	146.30 ± 4.10	24.66 ± 1.95	27.60 ± 0.28	6571.0 ± 423.42	7.40 ± 0.28
Kortowo Forest	18.85 ± 0.95	0.17 ± 0.01	20.60 ± 1.13	7.00 ± 0.57	217.00 ± 8.77	5.06 ± 0.42	25.20 ± 2.26	6439.1 ± 5.80	7.30 ± 0.71
State Road 51	37.50 ± 10.18	0.25 ± 0.07	24.20 ± 0.57	11.50 ± 0.99	158.00 ± 3.39	20.06 ± 1.50	32.60 ± 1.41	7689.9 ± 227.26	8.90 ± 0.14
City center	54.70 ± 0.82	0.14 ± 0.00	21.70 ± 0.14	11.30 ± 0.14	179.10 ± 0.71	36.72 ± 0.57	36.60 ± 0.28	7106.5 ± 81.32	9.60 ± 0.00
Average	32.91	0.18	21.28	11.36	170.54	20.24	28.86	6845.7	7.64
LSD	12.05	0.10	2.02	10.17	12.60	2.98	3.58	564.6	0.95

Significant at *P* ≤ 0.05

### Relations between trace elements in trees and soils

**Fig. 4:**
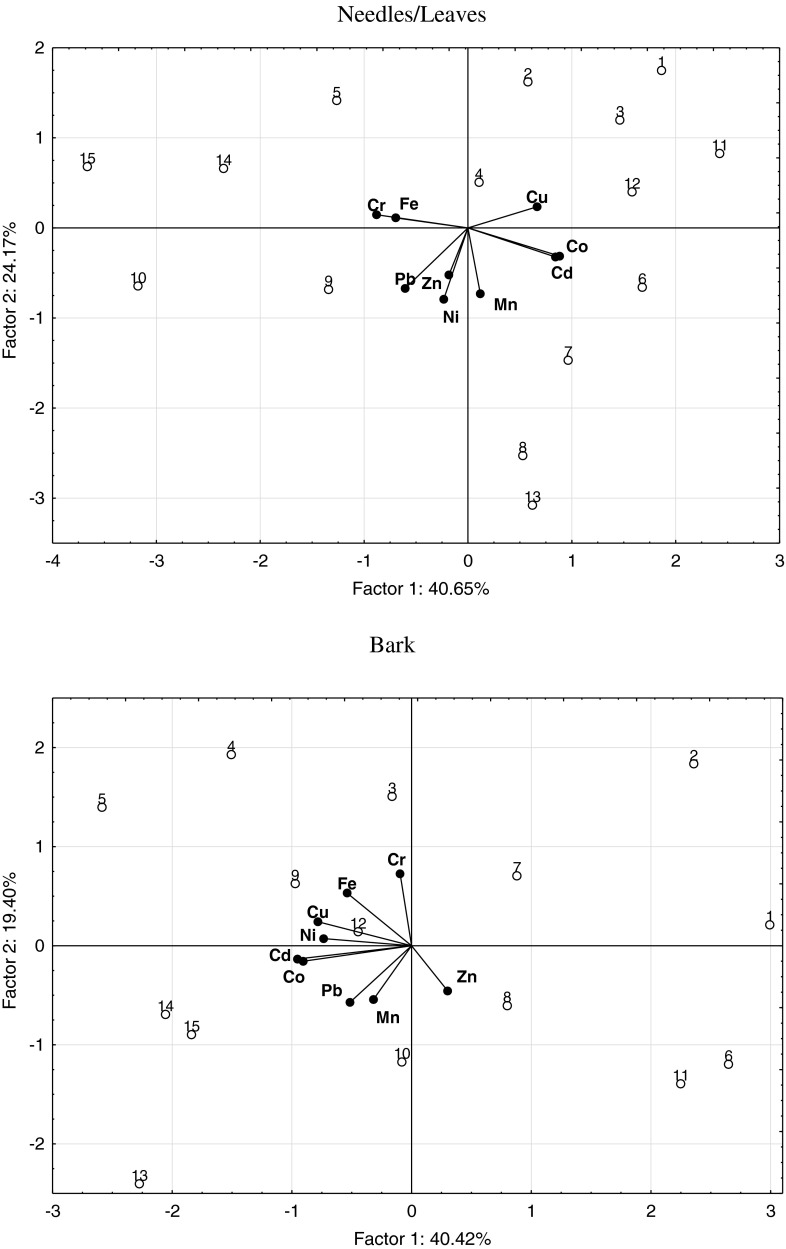
Content of elements in the trees illustrated with the PCA method. Key: vectors represent analyzed variable (content of Pb, Cd, Cr, Ni, Mn, Zn, Cu, Fe, and Co); points show soil samples with elements (*1*—Scots pine, MHPGP; *2*—Scots pine, Train–Dajtki; *3*—Scots pine, Kortowo Forest; *4*—Scots pine, State Road 51; *5*—Scots pine, City center; *6*—silver birch, MHPGP; *7*—silver birch, Train–Dajtki; *8*—silver birch, Kortowo Forest; *9*—silver birch, State Road 51; *10*—silver birch, City center; *11*—Norway maple, MHPGP; *12*—Norway maple, Train–Dajtki; *13*—Norway maple, Kortowo Forest; *14*—Norway maple, State Road 51; *15*—Norway maple, City center)

**Fig. 5:**
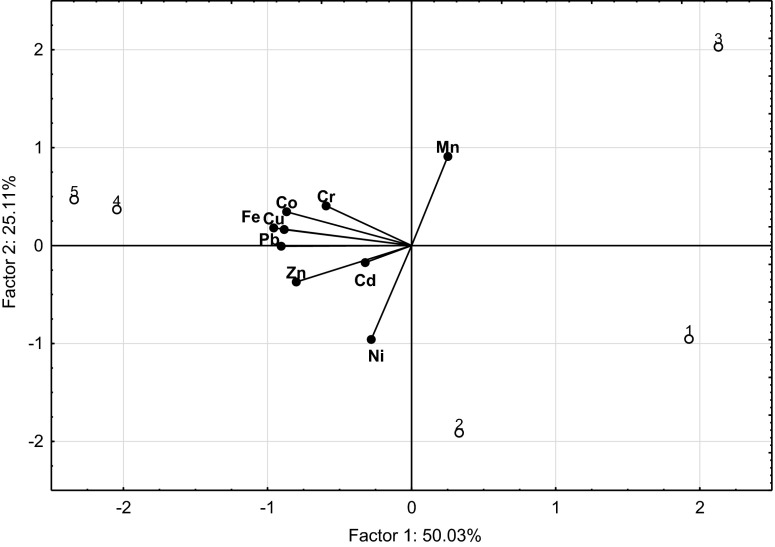
Content of elements in the soils illustrated with the PCA method. Key: vectors represent analyzed variable (content of Pb, Cd, Cr, Ni, Mn, Zn, Cu, Fe, and Co); points show soil samples with elements (*1*—MHPGP; *2*—Train–Dajtki; *3*—Kortowo Forest; *4*—State Road 51; *5*—City center)

The computed correlation coefficients as well as the PCA results (Table 6, Figs 4 and 5) implicate significant relationships between the following sets of elements: lead versus chromium, nickel, zinc, copper, and iron as well as cadmium versus chromium, manganese, copper, iron, and cobalt, or between chromium versus copper, iron, and cobalt, and between copper and iron versus cobalt in needles and leaves of trees. Regarding bark samples, significant correlations were determined between lead versus cadmium and cobalt; cadmium versus nickel, manganese, copper, and cobalt; nickel versus copper, iron, and cobalt; copper and iron versus cobalt. Significant correlations also appeared between the soil concentrations of lead versus zinc, copper, iron, and cobalt as well as chromium versus iron; nickel versus manganese; zinc versus copper; copper versus iron and cobalt; iron versus cobalt. Also, the content of manganese, copper, iron, and cobalt in soil was significantly correlated with the content of these elements in needles, leaves, and bark of trees (Table 7). The content of cadmium, manganese, zinc, copper, iron, and cobalt in needles and leaves was significantly correlated with the concentration of these elements in tree bark (Table 8). Low correlation coefficients computed for the other elements were a consequence of both their low content and relatively small differences between the locations where soil and plant material was sampled.

**Table 6:** Pearson’s simple correlation coefficients (*r*) between lead, cadmium, chromium, nickel, manganese, zinc, copper, and iron in the needles/leaves and bark of trees and in the soil

	Cd	Cr	Ni	Mn	Zn	Cu	Fe	Co
	Needles/leaves							
Pb	−0.256	0.372**	0.498**	0.347	0.512**	−0.435**	0.396**	−0.259
Cd		−0.687**	−0.049	0.414**	−0.034	0.503**	−0.552**	0.813**
Cr			0.097	−0.224	0.181	−0.370**	0.572**	−0.567**
Ni				0.333	0.309	−0.250	0.034	0.051
Mn					−0.024	−0.265	0.045	0.274
Zn						0.011	−0.195	0.006
Cu							−0.250	0.560**
Fe								−0.452**
	Bark							
Pb	0.374**	−0.216	0.358	0.167	0.285	0.141	0.100	0.578**
Cd		−0.068	0.483**	0.407**	−0.348	0.682**	0.263	0.914**
Cr			0.024	−0.170	−0.044	0.302	0.182	−0.040
Ni				0.012	0.101	0.425**	0.476**	0.524**
Mn					0.013	0.249	−0.171	0.278
Zn						−0.197	−0.333	−0.331
Cu							0.357	0.649**
Fe								0.368**
	Soil							
Pb	0.034	0.405	0.107	−0.168	0.869**	0.899**	0.645**	0.718**
Cd		0.189	0.194	−0.274	−0.057	0.225	0.479	0.239
Cr			−0.257	−0.039	0.076	0.414	0.817**	0.391
Ni				−0.640**	0.347	0.092	−0.145	−0.012
Mn					−0.408	0.014	−0.187	0.198
Zn						0.772**	0.432	0.599
Cu							0.732**	0.934**
Fe								0.685**

**P ≤ 0.05; *n* = 45

**Table 7:** Pearson’s simple correlation coefficients (*r*) between lead, cadmium, chromium, nickel, manganese, zinc, copper, and iron in the soil and in the needles/leaves and bark of trees

	Soil								
	Pb	Cd	Cr	Ni	Mn	Zn	Cu	Fe	Co
Needles/leaves	0.348	−0.050	0.326	−0.236	0.662**	0.194	−0.652**	0.392**	−0.689**
Bark	0.294	0.126	0.048	0.015	0.548**	−0.144	0.374**	0.563**	0.751**

**P ≤ 0.05; *n* = 45

**Table 8:** Pearson’s simple correlation coefficients (*r*) between lead, cadmium, chromium, nickel, manganese, zinc, copper, and iron in the bark and in the needles/leaves of trees

	Needles/leaves								
	Pb	Cd	Cr	Ni	Mn	Zn	Cu	Fe	Co
Bark	0.339	−0.392**	−0.113	0.154	0.971**	0.511**	−0.519**	0.381**	−0.494**

**P ≤ 0.05; *n* = 45

## Discussion

Trees are a good bioindicator and can therefore aid assessment of environmental pollution (Piczak et al. 2003, Sawidis et al. 2011). The following species most often serve this purpose: pine, spruce, fir, birch, beech, maple, horse-chesnut, linden, oak, poplar, and willow (Lamppu and Huttunen 2001, Piczak et al. 2003, Franiel and Więski 2005, Samecka-Cymermann et al. 2009, Sun et al. 2009, Sawidis et al. 2011). Currently, coniferous tree species are becoming more popular owing to their higher tolerance to pollution (Hermle et al. 2006). Differences in the content of heavy metals in individual tree species depending on the age of trees as well as the location of sampling sites were also attested by Kozik et al. (2014), Piczak et al. (2013). Piczak et al. (2003), who examined the content of iron in brittle willow, Norway maple, broad-leaved lime, and silver willow in two urban locations: a park in Wałbrzych and a site near a busy street in Wrocław, determined increased concentrations of iron. Such findings have been supported by the current investigations, in which the highest iron content was recorded in leaves of Norway maple growing in the town center and along Road 51. In our study, the major contributor to such high accumulation of iron in needles of Scots pine could have been trains running frequently on the railway track in Dajtki. According to Lamppu and Huttunen (2003), high iron content in pine needles can also be due to the proximity of ironworks. The iron content in needles of pines growing near ironworks was within the range of 430 to 740 mg kg^−1^. Malinowska (2010) as well as Sun et al. (2009) point to much higher accumulation of trace elements in trees growing in urban areas than in adjacent suburban or rural locations. Studies by Hrdlicka and Kula (2004) or Kozik et al. (2014) prove that leaves of Norway maple and silver birch are the most popular indicators of manganese accumulation. High zinc accumulation in different birch species has been suggested by Kopponen et al. (2001), Piczak et al. (2003), and Rosselli et al. (2003). The sensitivity of Norway pine to elevated zinc concentrations has been verified by Ivanov et al. (2011). According to Zverev et al. (2013), downy birch trees growing in north-western Russia, despite the vicinity of copper and nickel smelters, where the accumulation of emitted elements were relatively low. Palviainen et al. (2004) claim that a much higher content of iron is found in branches and leaves of trees than in their roots, although a reverse relationship for copper concentrations was observed by Kopponen et al. (2001) and Rosselli et al. (2003). Hrdlicka and Kula (2004) report that copper transport from roots to leaves is activated only when there is a deficit of this element. Sawidis et al. (2011) suggest that tree bark, like tree leaves, is a good bioindicator of environmental contamination.

The accumulation of the analyzed elements in soil reflects their content in needles, leaves, and bark of the examined trees, which is also claimed by Sawidis et al. (2011). In our study, the soil content of heavy metals was much higher than in the needles, leaves, and bark of the analyzed trees. This is also supported by the results of Chrzan (2013) concerning the content of lead, copper, and nickel in bark of Scots pine versus the content of the same elements in soil. Likewise, Sun et al. (2009) confirm that the content of lead and nickel in forest soils is lower than in urban areas. Rautio and Huttunen (2003) point to a higher zinc content in mineral than in organic soils. Kozlov et al. (2000) suggest that high soil pH can lead to higher availability of nickel for trees. Staszewski et al. (2012) conclude that a low sol pH affects the mobility of lead and zinc.

## Conclusions

The content of trace elements in needles of Scots pine, leaves of silver birch and Norway maple, and in bark of these trees depended on the location, tree species, and analyzed organ. The content of Fe, Mn, and Zn in needles, leaves, and bark of the examined tree species was significantly higher than that of the other elements. The highest average content of Fe and Mn was detected in leaves of Norway maple whereas the highest average content of Zn was found in silver birch leaves. The impact of such locations as the center of Olsztyn or roadside along Road 51 on the content of individual elements tended to be more pronounced than the influence of the other locations. The influence of the sampling sites on the content of trace elements in tree bark was less regular than the analogous effect in needles and leaves. Moreover, the relevant dependences were slightly different for Scots pine than for the other two tree species. The concentrations of heavy metals determined in the soil samples did not exceed the threshold values set in the Regulation of the Minister for the Environment, although the soil along Road 51 and in the center of Olsztyn typically had the highest content of these elements. There were also significant correlations between the content of some trace elements in soil and their accumulation in needles, leaves, and bark of trees.

## References

[CR1] Alagić SC, Šerbula SS, Tošić SB, Pavlović AN, Petrović JV (2013). Bioaccumulation of arsenic and cadmium in birch and lime from the Bor Region. Archives of Environmental Contamination and Toxicology.

[CR2] Chrzan A (2013). Contamination of soil and Pine bark by heavy metals in the selected forests. Ecological Chemistry and Engineering A.

[CR3] Franiel I, Więski K (2005). Leaf features of silver birch (*Betula pendula Roth*). Variability within and between two populations (uncontaminated vs Pb-contaminated and Zn-contaminated site). Trees.

[CR4] Hagen-Thorn A, Varnagiryte I, Nihlgard B, Armolaitis K (2004). Autumn nutrient resorption and losses in four deciduous forest tree species. Forest Ecology and Management.

[CR5] Hellsten S, Helmisaari H, Melin Y, Skovsgaard JP, Kaakinen S, Kukkola M, Saarsalmi A, Petersson H, Akselsson C (2013). Nutrient concentrations in stumps and coarse roots of Norway spruce, Scots pine and silver birch in Sweden, Finland and Denmark. Forest Ecology and Management.

[CR6] Hermle S, Günthardt-Goerg MS, Schulin R (2006). Effects of metal-contaminated soil on the performance of young trees growing in model ecosystems under field conditions. Environmental Pollution.

[CR7] Hrdlicka P, Kula E (2004). Changes in the chemical content of birch (*Betula pendula Roth*) leaves in the air polluted Krusne hory mountains. Trees.

[CR8] Ivanov YV, Savochkin YV, Kuznetsov VV (2011). Scots pine as a model plant for studying the mechanisms of conifers adaptation to heavy metal action: 1. Effects of continuous zinc presence on morphometric and physiological characteristics of developing Pine seedlings. Russian Journal of Plant Physiology.

[CR9] Kirchner P, Biondi F, Edwards R, McConnell JR (2008). Variability of trace metal concentrations in Jeffrey pine (*Pinus jeffreyi*) tree rings from the Tahoe Basin, California, USA. Journal of Forest Research.

[CR10] Kopponen P, Utriainen M, Lukkari K, Suntioinen S, Kärenlampi L, Kärenlampi S (2001). Clonal differences in copper and zinc tolerance of birch in metal-supplemented soils. Environmental Pollution.

[CR11] Kozik E, Golcz-Polaszewska M, Golcz A, Kuszak E, Kościelniak K (2014). Soils and plants the Nadolnik Park in Poznań. Part II. Content of microelements, cadmium and lead in soil and plants. Nauka Przyroda Technologie.

[CR12] Kozlov MV (2005). Sources of variation in concentrations of nickel and copper in mountain birch foliage near a nickel-copper smelter at Monchegorsk, north-western Russia: results of long-term monitoring. Environmental Pollution.

[CR13] Kozlov MV, Haukioja E, Bakhtiarov AV, Stroganov DN, Zimina SN (2000). Root versus canopy uptake of heavy metals by birch in an industrially polluted area: contrasting behaviour of nickel and copper. Environmental Pollution.

[CR14] Lamppu J, Huttunen S (2001). Scots pine needle longevity and gradation of needle shedding along pollution gradients. Canadian Journal of Forest Research.

[CR15] Lamppu J, Huttunen S (2003). Relations between Scots pine needle element concentrations and decreased needle longevity along pollution gradients. Environmental Pollution.

[CR16] Malinowska K (2010). Content of selected elements in the leaves growing in an urban agglomeration. Ecological Chemistry and Engineering A.

[CR17] Modrzewska B, Wyszkowski M (2014). Trace metal content in soils along State Road 51 (north-eastern Poland). Environmental Monitoring and Assessment.

[CR18] Modrzewska B, Kosiorek M, Wyszkowski M (2016). Content of some nutrients in Scots pine, silver birch and Norway maple in an urbanized environment. Journal of Elementology.

[CR19] Palviainen M, Finér P, Kurka AM, Mannerkoski H, Piirainen S, Starr M (2004). Release of potassium, calcium, iron and aluminium from Norway spruce, Scots pine and silver birch logging residues. Plant and Soil.

[CR20] Parzych A, Sobisz Z (2012). The macro- and microelemental content of *Pinus sylvestris* L. and *Pinus nigra* J.F. Arn. needles in *Cladonio-Pinetum* habitat of the Słowiński National Park. Forest Research Papers.

[CR21] Piczak K, Leśniewicz A, Żyrnicki W (2003). Metal concentrations in deciduous tree leaves from urban areas in Poland. Environmental Monitoring and Assessment.

[CR22] Rademacher P (2005). Nährelementgehalte in den Kompartimenten wichtiger Wirtschaftsbaumarten und deren Bedeutung für die Reststoffverwertung. Holz als Roh- und Werkstoff.

[CR23] Rautio P, Huttunen S (2003). Total vs. internal element concentrations in Scots pine needles along a sulphur and metal pollution gradient. Environmental Pollution.

[CR24] Regulation of the Minister of the Environment on 9 September 2002 on the standards of the soil quality and ground quality 1.09.2002. Dziennik Ustaw, Nr 165, 1359 (In Polish).

[CR25] Roitto M, Rautio P, Julkunen-Titto R, Kukkola E, Huttunen S (2005). Changes in the concentrations of phenolics and photosynthates in Scots pine (*Pinus sylvestris* L.) seedlings exposed to nickel and copper. Environmental Pollution.

[CR26] Rosselli W, Keller C, Boschi K (2003). Phytoextraction capacity of tree growing on a metal contaminated soil. Plant and Soil.

[CR27] Samecka-Cymerman A, Kolon K, Kempers AJ (2009). Short shoots of *Betula pendula Roth.* as bioindicators of urban environmental pollution in Wrocław (Poland). Trees.

[CR28] Sawidis T, Breuste J, Mitrovic M, Pavlovic P, Tsigaridas K (2011). Trees as bioindicator of heavy metal pollution in three European cities. Environmental Pollution.

[CR29] Staszewski T, Łukasik W, Kubiesa P (2012). Contamination of Polish national parks with heavy metals. Environmental Monitoring and Assessment.

[CR30] StatSoft, Inc. (2014). STATISTICA data analysis software system, version 12. www.statsoft.com.

[CR31] Sun FF, Wen DZ, Kuang YW, Li J, Zhang JG (2009). Concentrations of sulphur and heavy metals in needles and rooting soils of Masson pine (*Pinus massoniana* L.) trees growing along an urban-rural gradient in Guangzhou, China. Environmental Monitoring and Assessment.

[CR32] US-EPA Method 3051 (1994). Microwave assisted acid digestion of sediment, sludges, soils and oils.

[CR33] Zverev V, Kozlov M,V, Zvereva EL (2013). Changes in crown architecture as a strategy of mountain birch for survival in habitats disturbed by pollution. Science of the Total Environment.

